# Highlighting consensus among medical scientists increases public support for vaccines: evidence from a randomized experiment

**DOI:** 10.1186/s12889-015-2541-4

**Published:** 2015-12-03

**Authors:** Sander L. van der Linden, Chris E. Clarke, Edward W. Maibach

**Affiliations:** Department of Psychology and Woodrow Wilson School of Public Affairs, Princeton University, Princeton, NJ USA; Department of Communication, George Mason University, Fairfax, VA USA

## Abstract

**Background:**

A substantial minority of American adults continue to hold influential misperceptions about childhood vaccine safety. Growing public concern and refusal to vaccinate poses a serious public health risk. Evaluations of recent pro-vaccine health communication interventions have revealed mixed results (at best). This study investigated whether highlighting consensus among medical scientists about childhood vaccine safety can lower public concern, reduce key misperceptions about the discredited autism-vaccine link and promote overall support for vaccines.

**Methods:**

American adults (*N* = 206) were invited participate in an online survey experiment. Participants were randomly assigned to either a control group or to one of three treatment interventions. The treatment messages were based on expert-consensus estimates and either normatively described or prescribed the extant medical consensus: “90 % of medical scientists agree that vaccines are safe and that all parents should be required to vaccinate their children”.

**Results:**

Compared to the control group, the consensus-messages significantly reduced vaccine concern (*M* = 3.51 *vs. M* = 2.93, *p* < 0.01) and belief in the vaccine-autism-link (*M* = 3.07 *vs M* = 2.15, *p* < 0.01) while increasing perceived consensus about vaccine safety (*M* = 83.93 *vs M* = 89.80, *p* < 0.01) and public support for vaccines (*M* = 5.66 *vs M* = 6.22, *p* < 0.01). Mediation analysis further revealed that the public’s understanding of the level of scientific agreement acts as an important “gateway” belief by promoting public attitudes and policy support for vaccines directly as well as indirectly by reducing endorsement of the discredited autism-vaccine link.

**Conclusion:**

These findings suggest that emphasizing the medical consensus about (childhood) vaccine safety is likely to be an effective pro-vaccine message that could help prevent current immunization rates from declining. We recommend that clinicians and public health officials highlight and communicate the high degree of medical consensus on (childhood) vaccine safety when possible.

**Electronic supplementary material:**

The online version of this article (doi:10.1186/s12889-015-2541-4) contains supplementary material, which is available to authorized users.

## Background

Vaccines are one the most effective global public heath interventions, saving millions of lives every year [1]. Although childhood immunization rates in the U.S. are at a historic high [2] and there is widespread agreement among medical scientists about the safety and public health benefits of approved vaccines [3, 4], the number of American adults who report having heard “a great deal” about the disadvantages of vaccines for children has nearly doubled in the last 14 years (to 30 %), and over 52 % currently report being “unsure” whether certain vaccines cause autism [5, 6]. In addition, a recent national survey revealed that in a typical month, over 90 % of US physicians now frequently receive requests to “delay” child vaccines [7]. Growing concern about vaccines can erode public support and result in decreased immunization rates and recurrence of (preventable) life-threatening diseases [8] (e.g., the 2015 measles outbreak).

Systematic evaluations of public health communication strategies that focus on vaccine promotion range from being largely inconclusive about their general effectiveness [9, 10] (at best) to revealing that some messaging strategies may be counter-productive [11, 12] (at worst), especially among vaccine hesitant audiences [13]. One prominent issue is that media journalists frequently report arguments for and against vaccine safety in a “balanced” fashion that fails to emphasize the extant medical consensus [14]. This is important because recent research has found that communicating scientific consensus about vaccine safety attenuates perceptions of scientific uncertainty regarding vaccine risk [15, 16]. Moreover, for other contentious issues like climate change, public perception of the level of scientific agreement has shown to act as an important “gateway cognition,” influencing other key beliefs about the issue as well as support for action [17–20]. Highlighting consensus is thought to be particularly effective because it describes an important social norm within a community, which people often use as a heuristic to guide their beliefs and judgments on the issue [17, 18]. Consensus heuristics are efficient because they reduce the cost of individual learning by condensing a complex amount of information into a simple normative fact (e.g., 90 % of medical experts agree that vaccines are safe). This study examines whether highlighting the medical consensus on vaccine safety can increase public understanding of the scientific consensus, and, in turn, reduce misperceptions about the discredited autism-vaccine link and promote pro-vaccine attitudes, norms, and intentions.

## Method

We conducted a between-subject experiment in June of 2015. Participants (*N* = 206) were a diverse sample of American adults (56 % male, 18–75+, 45 % Democrat, see Appendix (Table 2) for a full description of the sample) recruited from Amazon Mechanical Turk (Mturk) – a platform which has shown to be more diverse and at least as reliable as other internet-based samples [21, 22]. Parental information was not recorded, as we focused on promoting science-based vaccine attitudes among adults broadly. Respondents were offered a small reward ($0.40) to complete an online survey in which they were randomly assigned to one of four experimental conditions; a *descriptive* norm condition (*n* = 59), a *prescriptive* norm condition (*n* = 60), a combination of the two (*n* = 44) or a control group (*n* = 43). Drawing on expert-survey estimates [3, 4], participants were shown a pie-chart which either stated that; “*90 % of medical scientists agree that vaccines are safe*” (descriptive), “*90 % of medical scientists agree that all parents should be required to vaccinate their children*” (prescriptive) or a combination of the two (see Additional file 1: Figure S1). Participants in the control group received no statement. Approval from Princeton’s Institutional Review Board (#7310) was obtained prior to the study. Participants also signed a written consent form.

After exposure to the treatment(s), all respondents answered the main survey questions. Perceived consensus was assessed with the following item; “to the best of your knowledge, what % of medical scientists agree that vaccines are safe?” (0 to 100 %). Perceived risk was assessed with the following item; “how concerned are you about the potential risks of vaccines? (1 = not concerned at all, 7 = very concerned). Endorsement of the autism link was assessed by asking people to what extent they agreed with the following statement; “there is scientific evidence for a causal link between vaccines and autism” (1 = strongly disagree - 7 = strongly agree). Public support for vaccines was assessed with 8 items, which were combined and averaged into a single measure to form a reliable index (cronbach’s α = 0.96), example items include; “I believe that vaccines are a safe and reliable way to avoid the spread of preventable diseases”, “I have already vaccinated my children or would do so if I had children” and “I would support policies that require people to vaccinate their children” (1 = strongly disagree - 7 = strongly agree). A full description of all measures used in the study is provided in the Appendix (Table 3). Results of the experiment were assessed through mean-comparisons (main effects) and mediation analysis (adjusted estimates) using STATA (StataCorp) v.13.

## Results

Observed differences in perceived consensus between the descriptive (*M* = 88.61, *SE* = 1.11), prescriptive (*M* = 90.62, *SE* = 1.11), and combined treatment (*M* = 90.27, *SE* = 1.06) variations were negligible; we therefore collapsed them into a single “consensus” treatment group. We conducted a Multivariate Analysis of Variance (MANOVA) to test for significant differences between the treatment conditions on the dependent variables (perceived scientific agreement, belief in the autism-vaccine link, risk perception and public support). Using Wilk’s criteria, we found a significant multivariate effect *F*(3, 202) = 5.05, *p* < 0.01, Wilk’s λ = 0.93. Adjusted univariate comparisons revealed a significant main effect (*p* < 0.01) for the consensus-message (compared to the control group) on all dependent variables (Table 1).

**Table 1:** Main effect of highlighting scientific consensus on dependent variables

Dependent variables	Mean consensus treatments	Mean control group	Cohen’s D
	(*n* = 163)	(*n* = 43)	
Perceived scientific agreement	**89.80** ^***^ (0.52)	83.93 (2.65)	0.60
Endorsement autism-vaccine link	**2.15** ^***^ (0.12)	3.07 (0.34)	0.55
Risk perception	**2.93** ^***^ (0.14)	3.51 (0.35)	0.31
Public support/attitudes	**6.21** ^***^ (0.09)	5.66 (0.24)	0.44

*Note*: Standard errors in parentheses. All mean comparisons significant at ^***^ *p* < 0.01 (bold face). Unequal variances assumed. Cohen’s *D* is a standardized measure of effect size. Values between 0.3 and 0.6 are generally considered to be “moderate” effect-sizes in behavioral science [25]

**Fig. 1:**
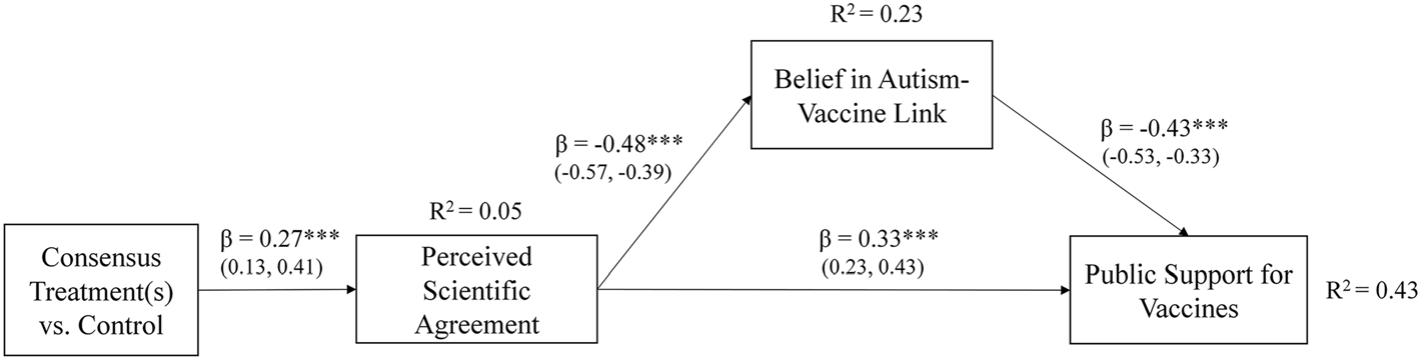
Perceived scientific agreement as a “gateway belief” mediation (path) model

We also estimated a mediation model to test whether the effect of the consensus-treatment messages on public support for vaccines is mediated by changes in the level of perceived scientific agreement on vaccine safety and (reduced) belief in the autism-vaccine link. The mediation model (Fig. 1) fit the data well. As expected, the model indicates that the effect of the consensus messages on public support and belief in the autism-link are fully mediated by changes in perceptions of scientific agreement. Perceived scientific agreement functions as an important “gateway” cognition by reducing belief in the autism-link (*negative effect*) and by increasing public support for vaccines (*positive effect*) both directly as well as indirectly. The indirect effect of perceived scientific agreement (B = 0.21, SE = 0.002) on public support via reduced endorsement of the autism link is substantial (approx. 38 % of the total effect is mediated). The model also reveals that belief in the autism-link (by itself) has a strong negative effect on public support for vaccines. Notably, almost half of the variation in public support (43 %) is explained by perceived scientific agreement and belief in the autism-link. Lastly, there was no significant interaction between the treatment-intervention(s) and political ideology on the dependent variables, the consensus messages shifted the views of liberals, moderates, and conservatives alike in line with the prevailing medical consensus.

## Discussion

While public concern over (childhood) vaccines is growing [5–7], recent attempts to communicate the health benefits of vaccines have failed to correct existing misperceptions and harness public support for the issue [9–13]. In contrast, our research shows that highlighting the degree of medical consensus increases perceived scientific agreement, which acts as a consequential “gateway” belief by promoting favorable public attitudes toward vaccination as well as by reducing perceived risk and belief in the (long discredited) autism-vaccine link. One plausible explanation for these promising results is that emphasizing consensus mitigates vaccine safety concerns in a way that does not require repeating a misinformation “myth” (e.g., mentioning a link between vaccines and autism). Research in cognitive psychology has shown that people are more likely to remember sticky “myths” than their “corrections” as revising pre-existing beliefs in light of new facts demands more cognitive effort [23]. Thus, while repeating a myth may simply reinforce existing beliefs, “setting the record straight” by emphasizing the high degree of medical consensus on vaccine safety avoids this dilemma [24]. The current study has a number of limitations. Particularly, our findings rely on a relatively small and non-representative sample of the American public. Although it is certainly possible that the typically younger and higher educated Amazon Turk participants are more reactive to the treatment than the general population, findings of this study are very much consistent with the results of communicating scientific consensus in other risk contexts [13–18] and extend prior pro-vaccine messaging interventions [13, 14] in a novel direction. In short, highlighting the (normative) consensus among medical scientists that vaccines are “safe” and that parents should be “required” to vaccinate their children is a promising public health communication strategy that may be able to protect current immunization rates from declining and limit the spread of otherwise preventable (life-threatening) diseases. Future research could extend these findings in several important ways. For example, the efficacy of medical consensus messaging could be assessed using (a) national samples of US adults, (b) among vaccine hesitant parents specifically and / or (c) in clinical field setting(s). One practical recommendation may include highlighting the degree of medical consensus about (childhood) vaccine safety in patient waiting rooms or in other clinical and public health settings (when appropriate).

## Conclusion

Results of this study suggest that highlighting the degree of medical consensus about (childhood) vaccine safety is likely to increase public support for vaccines both directly as well as indirectly by reducing influential misperceptions about the vaccine-autism link. In short, communicating the scientific consensus on vaccine safety is likely to be an effective pro-vaccine message that could help prevent current immunization rates from declining. We recommend that clinicians and public health officials emphasize the high degree of medical consensus on (childhood) vaccine safety whenever possible.

## Appendix

**Table 2:** Sample characteristics

Sample	(*N* = 216)
Demographic characteristics	
Gender (% male)	56
Female	44
Age (% 25–34)	41
18–24	12
35–44	23
45–54	12
55–64	9
65–74	2
75+	1
Education (% college degree or higher)	55
Less than High School	2
High School	7
Some College	36
Region % (South)	33
Midwest	28
Northeast	22
West	17
Party Affiliation (% Democrat)	45
Republican	28
Independent	27

*Note:* Modal category is reported first. Respondents were recruited from Amazon Mechanical Turk. Restrictions included location (United States) and a worker’s past approval rate (95 % or higher). Compared to the general U.S. national population, participants were more likely to be male, younger, higher educated, and self-identify as a Democrat

**Table 3:** Survey questions and descriptive statistics

Sample	Mean (S.D.)
Survey questions	
Perceived scientific agreement	
To the best of your knowledge, what % of medical scientists agree that vaccines are safe? (0–100 %).	88.39 (11.41)
Autism-vaccine link	
To what extent do you agree with the following statement; “there is scientific evidence for a causal link between vaccines and autism” (1 = Completely Disagree – 7 = Completely Agree).	2.43 (1.77)
Risk perception / concern	
How concerned are you about the potential risk of vaccines? (1 = I am not concerned at all, 7 = I am very concerned).	3.06 (1.92)
Public support index (Strongly Disagree = 1, Strongly Agree = 7).	6.20 (1.24)
I believe that vaccines are a safe and reliable way to avoid the spread of otherwise preventable diseases (*M* = 6.28, *SD* = 1.25).	
I have already vaccinated my children or would do so if I had children (*M* = 6.29, *SD* = 1.52).	
I would support policies that require people to vaccinate their children (*M* = 5.72, *SD* = 1.78).	
I believe that the health benefits of vaccines outweigh the risk of any potential negative side effects (*M* = 6.19, *SD* = 1.41).	
I believe that vaccines are important in maintaining and improving public health (*M* = 6.31, *SD* = 1.27).	
In the interest of public health, parents should simply be required to vaccinate their children (*M* = 5.75, *SD* = 1.72).	
More people ought to vaccinate themselves and their children (*M* = 6.20, *SD* = 1.48).	
I believe that vaccine refusal poses a risk to public health (*M* = 6.02, *SD* = 1.62).	

## Additional file

Additional file 1: Figure S1. Example treatment (Consensus-Message). (TIF 614 kb)
