# Erratum to: SWITCH: Highlighting consensus among medical scientists increases public support for vaccines: evidence from a randomized experiment

**DOI:** 10.1186/s12889-017-4198-7

**Published:** 2017-03-29

**Authors:** Sander L. van der Linden, Chris E. Clarke, Edward W. Maibach

**Affiliations:** 1 Department of Psychology and Woodrow Wilson School of Public Affairs, Princeton University, Princeton, NJ USA; 2 Department of Communication, George Mason University, Fairfax, VA USA

## Erratum

Following the publication of this article [1], it was brought to our attention that Table 2 of the Appendix contains a typographical error. The right column of the table incorrectly reads (*N* = 216) instead of (*N* = 206).

In addition, Table 3 of the Appendix contains some non-consequential rounding errors for the Mean (S.D.). The corrected table is provided below:

**Table 3:** Survey questions and descriptive statistics

Sample	Mean (S.D.)
Survey questions	
Perceived scientific agreement	
To the best of your knowledge, what % of medical scientists agree that vaccines are safe? (0% - 100%).	88.57 (10.14)
Autism-vaccine link	
To what extent do you agree with the following statement; “there is scientific evidence for a causal link between vaccines and autism” (1 = Completely Disagree – 7 = Completely Agree).	2.34 (1.71)
Risk perception/concern	
How concerned are you about the potential risk of vaccines? (1 = I am not concerned at all, 7 = I am very concerned).	3.05 (1.88)
Public support index (strongly disagree =1, strongly agree =7).	6.09 (1.32)
I believe that vaccines are a safe and reliable way to avoid the spread of otherwise preventable diseases (*M* = 6.29, *SD* = 1.20).	
I have already vaccinated my children or would do so if I had children (*M* = 6.29, *SD* = 1.52).	
I would support policies that require people to vaccinate their children (*M* = 5.73, *SD* = 1.78).	
I believe that the health benefits of vaccines outweigh the risk of any potential negative side effects (M = 6.16, SD = 1.38).	
I believe that vaccines are important in maintaining and improving public health (M = 6.31, SD = 1.25).	
In the interest of public health, parents should simply be required to vaccinate their children (M = 5.76, SD = 1.70).	
More people ought to vaccinate themselves and their children (M = 6.21, SD = 1.48).	
I believe that vaccine refusal poses a risk to public health (M = 6.0, SD = 1.62).	

The above errors do not influence the findings and conclusions presented in the article [1].
