# Supplementary figures and images for: Highlighting consensus among medical scientists increases public support for vaccines: evidence from a randomized experiment
Source: BMC Public Health. 2015 Dec 3;15:1207. doi: 10.1186/s12889-015-2541-4 (PMC4669673; doi:10.1186/s12889-015-2541-4)

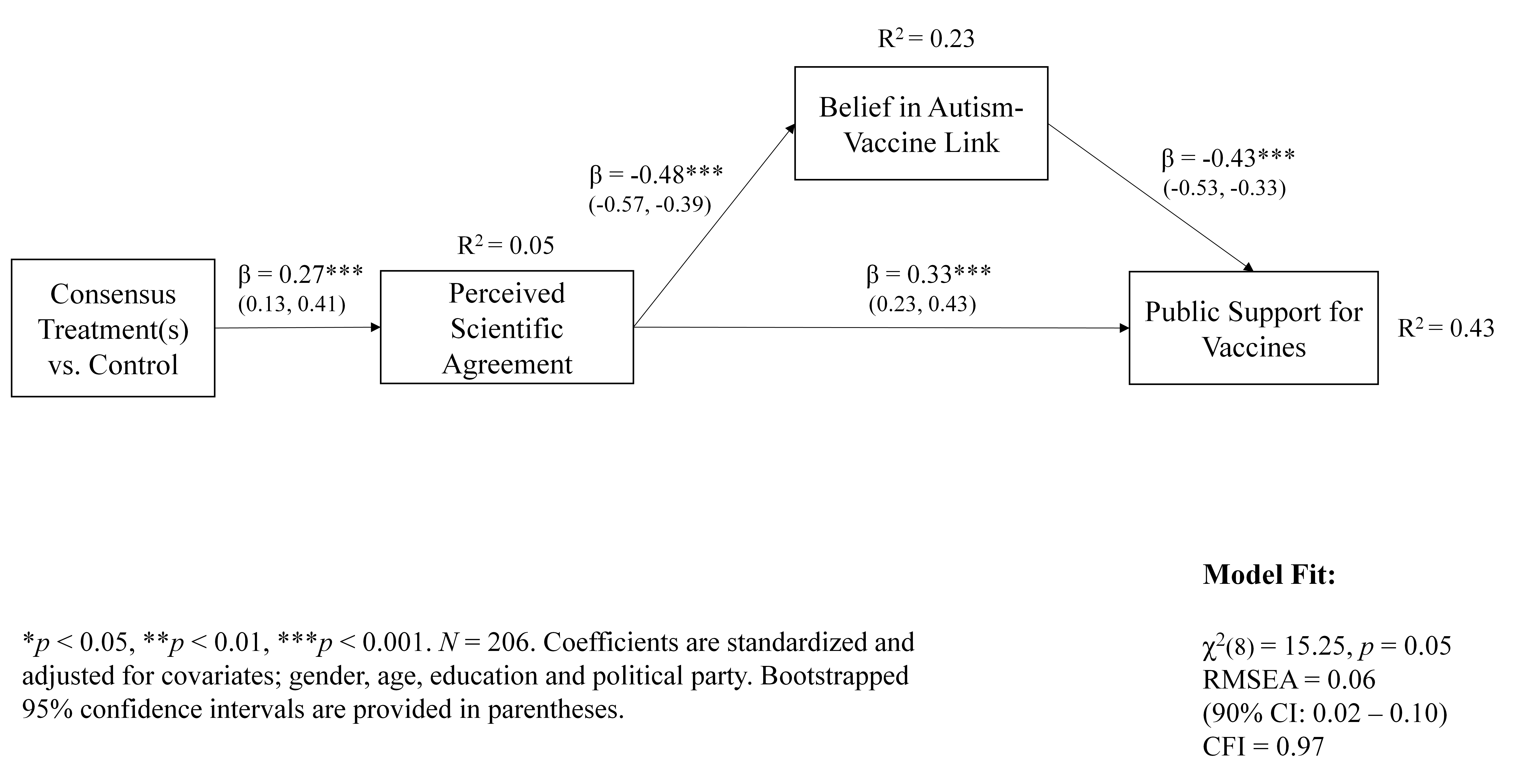

Supplement: Additional file 1: Figure S1. — Example treatment (Consensus-Message). (TIF 614 kb) [file 12889_2015_2541_MOESM1_ESM.tif]
